# Clinical Trial of Autologous Dendritic Cell Administration Effect on Water Molecule Diffusion and Anti-Inflammatory Biomarkers in Diabetic Kidney Disease

**DOI:** 10.3390/cimb46120822

**Published:** 2024-12-04

**Authors:** Paulus Stefanus Dimu, Aziza Ghanie Icksan, Farhat, Jonny, Bhimo Aji Hernowo, Terawan Agus Putranto

**Affiliations:** 1 Faculty of Medicine, Dentistry, and Health Science, Universitas Prima Indonesia, Medan 20118, Indonesia; paulusdimu@gmail.com (P.S.D.); farhatmedan@gmail.com (F.); jonny@unprimdn.ac.id (J.); hernowobhimo@gmail.com (B.A.H.); terawanagusputranto@unprimdn.ac.id (T.A.P.); 2 Department of Radiology, Slamet Riyadi Army Hospital, Surakarta 57148, Indonesia; 3 Faculty of Medicine, Universitas Pembangunan Nasional “Veteran” Jakarta, Jakarta 12450, Indonesia; 4 Faculty of Military Medicine, Indonesia Defense University, Bogor 16810, Indonesia; 5 Nephrology Division, Department of Internal Medicine, Gatot Soebroto Central Army Hospital, Jakarta 10410, Indonesia; 6 Indonesia Army Cellcure Center, Gatot Soebroto Central Army Hospital, Jakarta 10410, Indonesia; 7 Department of Radiology, Gatot Soebroto Central Army Hospital, Jakarta 10410, Indonesia

**Keywords:** diabetic kidney disease, diffusion-weighted MRI, apparent diffusion coefficient, intracellular adhesion molecule-1, transforming growth factor-β, autologous dendritic cells

## Abstract

Diabetic kidney disease (DKD) significantly increases mortality, with patients facing a fourfold risk of death within ten years. Chronic inflammation, marked by transforming growth factor-β (TGF-β) and intracellular adhesion molecule-1 (ICAM-1) activity, contributes to kidney damage and fibrosis. This study investigates the effect of autologous dendritic cells on inflammation and kidney function, focusing on apparent diffusion coefficient (ADC), TGF-β, and ICAM-1 levels. This quasi-experimental clinical trial involved 22 DKD patients at Gatot Soebroto Army Hospital. Patients received autologous dendritic cell injections. Baseline and post-intervention magnetic resonance imaging (MRI) scans measured ADC values, and ICAM-1 and TGF-β levels were analyzed. Post intervention, the median ADC decreased from 1.75 mm^2^/s to 1.64 mm^2^/s (*p* = 0.223). ICAM-1 levels increased significantly in females (*p* = 0.04) but not in males (*p* = 0.35). No significant changes were found in TGF-β levels (*p* = 0.506). ADC changes were statistically insignificant and did not correlate with CKD severity. ICAM-1 increases in females correlated with improved creatinine levels, suggesting kidney function improvement. Autologous dendritic cell therapy revealed potential gender-specific immune responses but showed limited overall biomarker improvements. Further studies are required to validate its therapeutic value.

## 1. Introduction

Diabetic kidney disease (DKD) is a severe microvascular complication in people with diabetes, which can develop into chronic kidney disease (CKD). Based on Global Burden of Disease data, approximately 27% of DM patients experience DKD, which contributes to 40% of total CKD cases [1]. In Indonesia, DKD is the second largest cause of stage 5 CKD after hypertension, with 28% of CKD patients exhibiting DKD [2]. DKD significantly increases the risk of death. Patients have a four times greater risk of dying within ten years than those without DKD [3]. In addition, the management of DKD imposes a high health cost burden, with approximately 10% of the total catastrophic disease costs in Indonesia allocated to the management of chronic kidney disease in 2021 [4].

Chronic inflammation in diabetes plays a vital role in the development of DKD. This inflammation releases reactive oxygen species (ROS) and inflammatory mediators that damage kidney tissue, triggering fibrosis and changes in kidney structure [5]. The adhesion molecule Intercellular Adhesion Molecule-1 (ICAM-1) plays a role in the progression of DKD by facilitating leukocyte adhesion and infiltration into renal tissue, which in turn increases albuminuria and renal inflammation [6,7]. Studies show that ICAM-1 expression correlates with the degree of albuminuria in DKD patients, making it an essential indicator for diagnosis. Structural changes in the kidney due to fibrosis can also be identified radiologically through diffusion-weighted magnetic resonance imaging (DW-MRI). A decrease in the apparent diffusion coefficient (ADC) value produced by DW-MRI indicates microvascular damage in the kidney, which is helpful in detecting DKD early [8].

Although standard therapies such as renin–angiotensin system (RAS) inhibitors and sodium-glucose cotransporter 2 (SGLT2) inhibitors have been shown to slow the rate of progression of DKD and reduce the severity of microalbuminuria, the results are still unsatisfactory, especially in patients with normal blood pressure or without microalbuminuria. The combination of the two therapies has also not completely stopped the progression of DKD [9,10]. Therefore, there is an urgent need to develop additional therapies that can target the underlying pathogenesis of DKD, namely chronic low-grade inflammation. One potential therapy being investigated is the use of autologous dendritic cells. These dendritic cells, which are part of the innate immune system, can suppress the inflammatory response. Research suggests that autologous dendritic cells can be used as anti-inflammatory agents in various diseases, including arthritis, kidney disease, and autoimmune diseases [11,12,13].

Dendritic cells (DCs) can reduce inflammation through various mechanisms that promote immune tolerance and homeostasis. They can differentiate into tolerogenic dendritic cells (tolDCs), which suppress excessive immune responses and help the immune system recognize self-antigens, preventing autoimmune reactions. DCs also produce anti-inflammatory cytokines like interleukin (IL)-10 and transforming growth factor-β (TGF-β), which inhibit pro-inflammatory cells and promote the activation of regulatory T cells (Tregs) that further limit inflammation. Additionally, DCs resist maturation in the presence of anti-inflammatory signals, interact with other immune cells like NK cells and macrophages to fine-tune the immune response, and can be used in therapies to induce targeted immune responses without triggering widespread inflammation [14,15]. Autologous DCs have also shown potential for reducing inflammation in autoimmune diseases like Systemic Lupus Erythematosus (SLE), with a case study demonstrating significant clinical improvement after DC-based therapy. The treatment involved using DCs derived from the patient’s blood and maturated to stimulate an immune response, leading to improvements in symptoms such as joint pain and muscle weakness [16]. With the potential for ex vivo generation of anti-inflammatory DCs, these cells offer promising therapeutic possibilities for treating inflammatory and autoimmune diseases, including diabetic kidney disease [17]. Dendritic cells’ anti-inflammatory potential can be an effective adjuvant therapy to suppress inflammation in DKD, prevent further damage to kidney structure and function, and slow disease progression. This study aims to determine the effect of autologous dendritic cell administration in reducing inflammation in DKD and slowing disease progression, as measured by ADC, TGF-β, and ICAM-1 parameters.

## 2. Materials and Methods

### Study Design

This study is a quasi-experimental clinical trial with a pre-test and post-test design, which aims to evaluate changes in ADC in DKD patients after administration of autologous dendritic cells. This study was conducted at Gatot Soebroto Army Central Hospital (RSPAD GS). The Health Research Ethics Committee of RSPAD GS approved the study. Before the start of the study, all subjects were given clear information about the study’s purpose, procedures, risks, and benefits. The subjects were allowed to ask questions, and after fully understanding, they signed an informed consent form as a sign of their agreement to participate in this study. Strict research ethics were applied to protect the subjects’ rights during the clinical trial. The study subjects consisted of DKD patients who met the inclusion criteria and were outpatients at the polyclinic of RSPAD GS during April 2024. The sampling technique used a consecutive sampling method, where subjects who met the requirements would be included in the study sequentially until the minimum quota of 20 subjects was met. With this number of subjects, it is expected to detect significant changes in ADC values and substantial modifications in TGF-β and ICAM-1.

The inclusion criteria for this study required participants to be over 18 years old, able to understand and sign informed consent, and capable of complying with the research procedures. They also had to meet the 2021 PERKENI diagnostic criteria for Type 2 Diabetes Mellitus (DM), such as fasting plasma glucose of ≥126 mg/dL (normal < 126 mg/dL), blood glucose ≥200 mg/dL (normal < 200 mg/dL) two hours after a glucose tolerance test, or HbA1c ≥ 6.5% (normal: < 5.7, prediabetes: 5.7–6.4). Additionally, participants needed an estimated glomerular filtration rate (eGFR) ≥30 mL/min/1.73 m^2^ (normal ≥ 90 mL/min/1.73 m^2^) and a urine albumin-to-creatinine ratio (UACR) ≥30 mg/g (normal < 30 mg/g). Exclusion criteria included recent immunosuppressive treatment, kidney diseases like polycystic kidney disease or lupus nephritis, other proteinuria-causing conditions, a positive pregnancy test, immunodeficiency disorders (HIV, HCV, HBV), cancer under active treatment (except hormonal therapy), and thromboembolic history. Participants with physical or mental disabilities, uncontrolled hypertension (SBP > 170 mmHg, DBP >100 mmHg), or BMI over 40 kg/m^2^ were also excluded.

A 40-milliliter blood sample was collected from each participant, and Peripheral Blood Mononuclear Cells (PBMCs) were isolated using density gradient centrifugation with Ficoll-Paque. These PBMCs were then cultured with Granulocyte–Macrophage Colony-Stimulating Factor (GM-CSF) and interleukin-4 (IL-4) for five days to generate immature DCs. Afterward, the cells were incubated with an antigen for two additional days to induce maturation. The quantity of dendritic cells administered varied between participants based on the yield from their individual blood samples, with no adjustments to standardize cell counts. Consequently, the total dose administered ranged from approximately 0.5 to 8 million DCs per participant, depending on the specific yield from each sample.

In the first week, a screening phase was conducted to determine the eligibility of subject participation according to predetermined criteria. After that, subjects underwent a baseline examination, which included an MRI to assess ADC and initial laboratory tests. Blood was drawn to prepare autologous dendritic cells that would later be used in the injection process. After that, in the intervention phase, autologous dendritic cells were injected into each subject. After the injection, an evaluation of ADC was conducted in week 4. In addition, laboratory evaluation and inflammatory biomarkers, TGF-β and ICAM-1, were also performed at baseline and four weeks after injection to see changes in the inflammatory response. Both biomarkers were measured using sandwich Enzyme-Linked Immunosorbent Assay (ELISA) kits (Reed Biotech Ltd., Wuhan, China), known for their high sensitivity and specificity in detecting these proteins in serum.

ADC measurements on renal MRI were performed using ADC maps generated from DWI (diffusion-weighted imaging) at a value of b = 1000 s/mm^2^. The senior radiographer defined three ROIs (Regions of Interest) in each kidney in the parenchymal area without distinguishing between the cortex or medulla. Each ROI was analyzed to obtain the ADC value in mm^2^/s.

The criteria for choosing between parametric and non-parametric analysis in this study were based on the distribution of the data. The Shapiro–Wilk normality test was initially performed to assess whether the data followed a normal distribution. If the data were found to be normally distributed, this study applied parametric analysis, specifically the Paired *t*-test, to compare the differences before and after the intervention. This test assumes that the data follow a normal distribution and that the differences between paired observations are symmetrically distributed.

If the data were found to be non-normally distributed (i.e., did not meet the assumptions for parametric testing), non-parametric analysis was used. In this case, the Wilcoxon Signed-Rank Test was employed to compare the before and after intervention data. The Wilcoxon test does not require the assumption of normality and is appropriate for ordinal or skewed data.

To assess relationships between continuous variables, Pearson correlation analysis was used when the data were normally distributed, as it assumes a linear relationship and normality between variables. If the data were non-normally distributed, Spearman’s rank correlation was applied, as it is a non-parametric test that does not assume normality and is suited for ordinal or skewed data.

**Figure 1:**
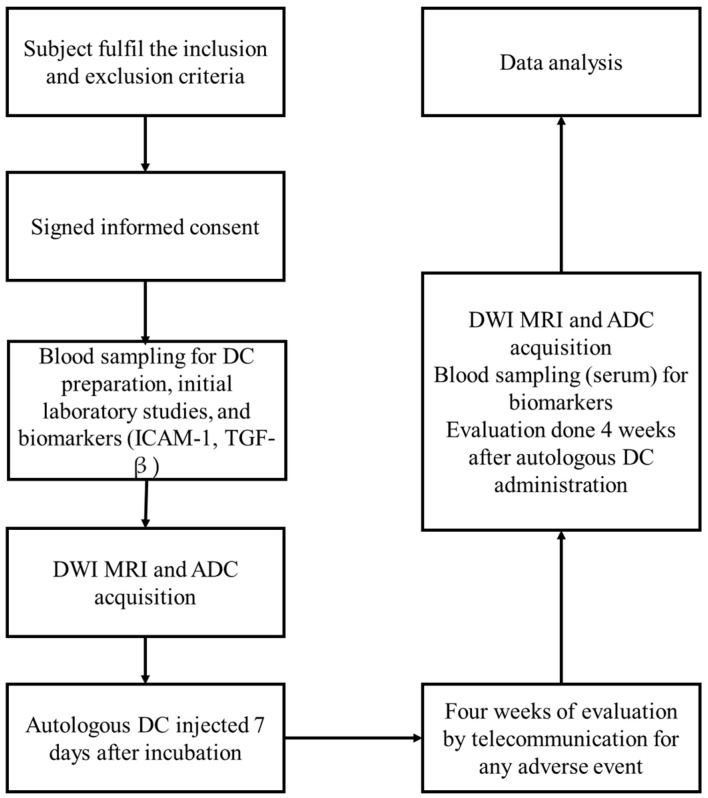
Study flowchart.

For subgroup analysis, if comparing two independent groups with normally distributed data, the Independent Samples *t*-test was used. If the data in the subgroups were non-normally distributed, the Mann–Whitney U test (a non-parametric equivalent of the Independent Samples *t*-test) was used to compare differences between the groups (Figure 1). Statistical analyses were done using IBM SPSS Statistics 25 (IBM, Armonk, NY, USA).

## 3. Results

### 3.1. Subject Characteristics

After screening based on the inclusion criteria, 156 patients met the requirements, while 510 patients did not. Of the 156 patients who met the criteria, 23 agreed to become research subjects, while 133 patients refused. Of the 23 consenting subjects, 22 underwent this study, but 1 was excluded due to meeting the exclusion criteria.

This study included 22 subjects who met the inclusion and exclusion criteria and completed the entire study procedure. The average age of the subjects was 64 years, with 9 (41%) men and 13 (59%) women. Hypertension was the most common comorbidity experienced by 20 subjects (90.9%). Most subjects had a body mass index in the overweight category, with nine subjects (40.9%). Most subjects also used insulin, as many as 17 subjects (77.3%) (Table 1).

### 3.2. Correlation of ADC and eGFR

**Figure 2:**
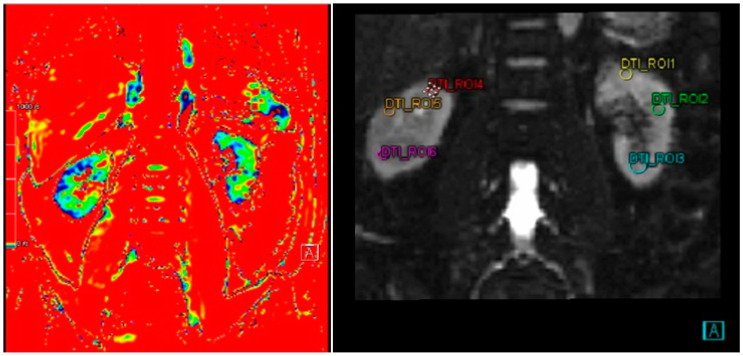
DWI image and ADC measurement on six different ROIs. A coronal DW-MRI and ADC map were obtained from a 68-year-old male volunteer with an eGFR of 70.65. Three ROIs were placed in the upper pole, middle portion, and lower pole of both kidneys. The mean ADC values for the left and right kidneys were 2.695 × 10^−3^ mm^2^/s and 2.877 × 10^−3^ mm^2^/s, respectively.

The ADC values, representing the diffusion of water molecules within kidney tissue, were recorded from six ROIs (Regions of Interest) across the right and left kidneys (Figure 2). For the left kidney, pre-intervention ADC values ranged from 1.734 to 1.779 × 10^3^ mm^2^/s across ROI 1 to ROI 3, while post-intervention values ranged from 1.529 to 1.736 × 10^3^ mm^2^/s. A statistically significant reduction was observed in ROI 1 (*p* = 0.017), whereas no significant changes were found in ROI 2 (*p* = 0.910) or ROI 3 (*p* = 0.615). Similarly, in the right kidney, pre-intervention ADC values ranged from 1.818 to 1.951 × 10^3^ mm^2^/s across ROI 4 to ROI 6. Post-intervention values showed slight decreases, ranging from 1.675 to 1.824 × 10^3^ mm^2^/s. No statistically significant changes were observed in ROI 4 (*p* = 0.277), ROI 5 (*p* = 0.101), or ROI 6 (*p* = 0.709) (Table 2).

**Table 2:** Change in ADC on each ROI.

ADC Values (×10^3^ mm^2^/s)	Lef Kidney			Right Kidney		
	ROI 1	ROI 2	ROI 3	ROI 4	ROI 5	ROI 6
Pre	1.734	1.740	1.779	1.839	1.951	1.818
Post	1.529	1.728	1.736	1.675	1.824	1.773
*p*-value	0.017	0.910	0.615	0.277	0.101	0.709

Hypothesis test using Wilcoxon: ROI 1: left kidney, upper region; ROI 2: left kidney, middle region; ROI 3: left kidney, lower region; ROI 4: right kidney, upper region; ROI 5: right kidney, middle region; ROI 6: right kidney, lower region.

In the group with eGFR > 60 mL/min/1.73 m^2^, the correlation value between ADC and eGFR was −0.267, indicating a weak negative correlation between the two variables. However, the *p*-value of 0.457 indicates that this relationship is not statistically significant, so it cannot be concluded that there is a meaningful relationship between ADC and eGFR in this group (Table 3).

**Table 3:** ADC and eGFR correlation based on eGFR level.

	Correlation of ADC and eGFR	*p*-Value *
eGFR > 60 mL/min/1.73 m^2^	−0.267	0.457
eGFR < 60 mL/min/1.73 m^2^	0.079	0.806

* Correlation test using Spearman’s rank correlation.

In contrast, in the group with eGFR < 60 mL/min/1.73 m^2^, the correlation value between ADC and eGFR was 0.079, indicating a very weak positive correlation between the two variables. The *p*-value of 0.806 also suggested that this relationship was not statistically significant. Therefore, there was no substantial evidence to suggest a meaningful relationship between ADC and eGFR in this group.

### 3.3. Changes in ADC

**Figure 3:**
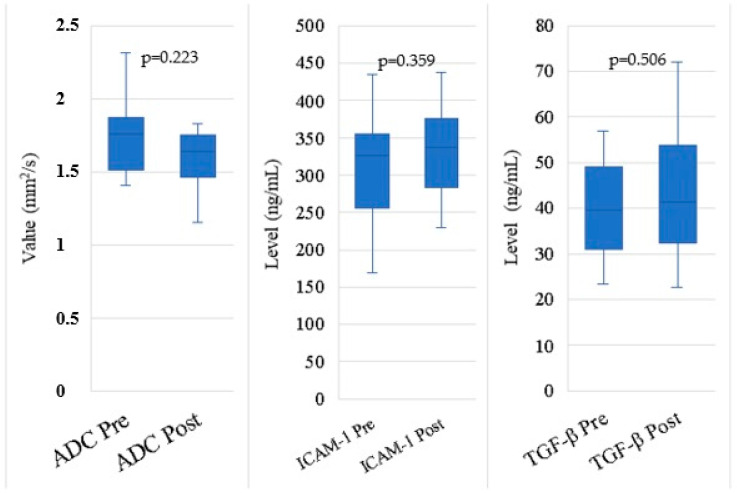
Change in ADC, ICAM-1, and TGF-β.

In this study, ADC values were measured before and after the intervention. The median ADC value before the intervention was 1.75 mm^2^/s, with a first to third quartile range (Q1–Q3) of 1.51 to 1.87 mm^2^/s. After the intervention, the median ADC decreased to 1.64 mm^2^/s, with a quartile range of 1.46 to 1.75 mm^2^/s. This decrease indicates a change in ADC values post intervention. The normality test using Shapiro–Wilk showed that the ADC data before the intervention were normally distributed with a *p*-value of 0.223. Furthermore, hypothesis testing using the Wilcoxon Signed Rank Test yielded a *p*-value of 0.003, indicating a statistically significant difference between ADC values before and after the intervention (Table 4, Figure 3).

**Table 4:** Change in ADC, ICAM-1, and TGF-β.

Variables	Median (Q1–Q3)	*p*-Value Hypothesis Test
ADC Pre	1.75 mm^2^/s (1.51–1.87)	0.223 ^a^
ADC Post	1.64 mm^2^/s (1.46–1.75)	
ICAM-1 Pre	325.6 ng/mL (256–355.3)	0.359 ^b^
ICAM-1 Post	336.7 (283.8–375.3)	
TGF-β Pre	39.55 ng/mL (30.9–49)	0.506 ^a^
TGF-β Post	41.35 ng/mL (32.4–53.7)	

^a^ Hypothesis testing using the Wilcoxon Signed Rank Test; ^b^ hypothesis testing using the Paired *t*-test.

### 3.4. Changes in ICAM-1 and TGF-β Levels

For ICAM-1 parameters, the median value before autologous dendritic cell administration was 0.32 ng/mL with an IQR of 0.25–0.35, and these data were typically distributed based on the Shapiro–Wilk test (*p* = 0.668). Hypothesis testing using the Paired *t*-test showed no significant difference between pre- and post-administration values of autologous dendritic cells (*p* = 0.359). Post administration of autologous dendritic cells, the median ICAM-1 value increased to 0.33 ng/mL with an IQR of 0.28–0.37 and a normality test *p*-value of 0.798.

In the TGF-β parameter, the median value before autologous dendritic cell administration was 39.55 ng/mL with an IQR of 30.9–49. The normality test showed that the data were normally distributed (*p* = 0.36). Hypothesis testing using the Wilcoxon Signed Rank Test showed no significant difference between pre- and post-administration values of autologous dendritic cells (*p* = 0.506). After the administration of autologous dendritic cells, the median TGF-β value increased to 41.35 ng/mL with an IQR of 32.4–53.7 and a normality test *p*-value of 0.346.

### 3.5. Correlation of ADC, TGF-β, and VCAM

**Figure 4:**
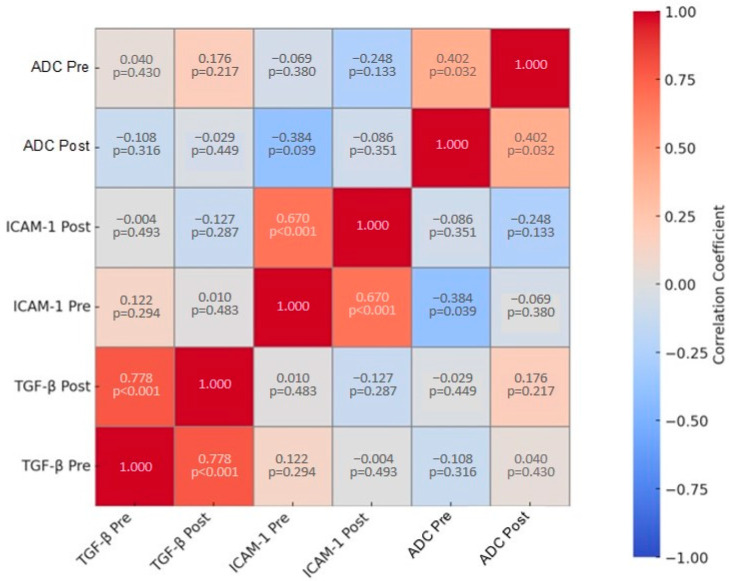
Heat map of variables’ correlation coefficients.

Correlations involving ICAM-1 show weaker relationships, with coefficients such as 0.122 (ICAM-1 Pre with TGF-β Pre) and −0.004 (ICAM-1 Post with TGF-β Pre), both with non-significant *p*-values. The mean pre-ADC values indicate a significant correlation with ICAM-1 Pre (−0.384, *p*-value 0.039), while other mean ADC comparisons exhibit non-significant *p*-values (Table 5, Figure 4). Overall, the table summarizes the relationships between the variables, highlighting the varying strengths and significances of these correlations.

**Table 5:** ADC, ICAM-1, and TGF-β level correlation.

	TGF-β Pre	TGF-β Post	ICAM-1 Pre	ICAM-1 Post	Mean ADC Pre	Mean ADC Post
TGF-β Pre	1.000	0.778	0.122	−0.004	−0.108	0.040
*p*-value		0.000	0.294	0.493	0.316	0.430
TGF-β Post	0.778	1.000	0.010	−0.127	−0.029	0.176
*p*-value	0.000		0.483	0.287	0.449	0.217
ICAM-1 Pre	0.122	0.010	1.000	0.670	−0.384	−0.069
*p*-value	0.294	0.483		0.000	0.039	0.380
ICAM-1 Post	−0.004	−0.127	0.670	1.000	−0.086	−0.248
*p*-value	0.493	0.287	0.000		0.351	0.133
Mean ADC Pre	−0.108	−0.029	−0.384	−0.086	1.000	0.402
*p*-value	0.316	0.449	0.039	0.351		0.032
Mean ADC Post	0.040	0.176	−0.069	−0.248	0.402	1.000
*p*-value	0.430	0.217	0.380	0.133	0.032	

Further analysis was performed to determine the effect of autologous dendritic cell administration on ADC values, TGF-β levels, and ICAM-1 by looking at the correlation between variables. Each variable was compared to the ratio of its value from post administration divided by the value pre-administration of autologous dendritic cells. The median of each variable ratio was determined. ADC, TGF-β, and ICAM-1 ratios above the median value were considered to have worsened, and vice versa, ratios below the median value were considered to have improved. Pearson’s correlation test showed that the ICAM-1 ratio and ADC ratio had a significant negative correlation with *p* = 0.010. At the same time, the TGF-β ratio and ADC ratio were positively correlated but not significant, with *p*-value = 0.479 (Table 6).

**Table 6:** ADC, ICAM-1, and TGF-β ratio correlation.

	ADC Ratio	
	Correlation	*p*-Value *
ICAM-1 Ratio	−0.490	0.010
TGF-β ratio	−0.12	0.479

* Correlation test using Spearman’s rank correlation.

### 3.6. Changes in ADC Values, Serum Creatinine Levels, ICAM-1, and TGF-β in Gender Subgroups

The mean change in ICAM-1 levels in men was 18.23 ± 55.1 with a *p*-value of 0.35, indicating that this change was not statistically significant (*p* > 0.05). On the other hand, the mean change in ICAM-1 levels in women was −31.77 ± 49.8 with a *p*-value of 0.04, indicating that this change was statistically significant (*p* < 0.05) (Table 7). These results suggest a significant difference in the shift in ICAM-1 levels in women after the intervention, while there was no significant change in men.

**Table 7:** ADC values, serum creatinine levels, ICAM-1, and TGF-β level changes in gender subgroups.

	Paired *t*-Test		Wilcoxon Signed Rank Test					
	Mean ICAM-1 (Post–Pre)	*p*-Value	Z TGF-β	*p*-Value	Z ADC	*p*-Value	Z Creatinine	*p*-Value
Men	−18.23 ± 55.1	0.35	−1.244	0.214	−0.533	0.594	−0.59	0.953
Women	31.77 ± 49.8	0.04	−0.105	0.917	−0.943	0.345	−2.030	0.042

Changes in TGF-β levels and ADC values before and after the administration of autologous dendritic cells in men and women showed no significant differences. In men, the *p*-value for changes in TGF-β was 0.214, and for ADC, it was 0.594, which indicated no significant change after autologous dendritic cell administration. Similarly, in women, the *p*-value for TGF-β was 0.917, and for ADC, it was 0.345, indicating no significant change in either variable after autologous dendritic cell administration. Creatinine levels in both groups also showed different results. Although the trends of both groups were similar, there was a decrease in creatinine levels after autologous dendritic cell administration. Still, there was a significant decrease in creatinine in the female group (Table 7).

### 3.7. Changes in ADC Values, Serum Creatinine Levels, ICAM-1, and TGF-β Based on Cardiovascular Comorbidities

Table 8 presents information focused on two specific groups. Those with cardiovascular comorbidities, including heart failure, coronary heart disease, and acute coronary syndrome, and those with resistant hypertension are defined as patients requiring three or more anti-hypertensive medications without achieving the target blood pressure. The results indicate that there were no significant differences in ADC, ICAM-1, TGF-β, or creatinine levels between patients with and without heart disease comorbidities at both pre-and post-autologous DC administration. Similarly, the analysis revealed no significant differences in these biomarkers between patients with and without resistant hypertension. The findings suggest that the presence of cardiovascular comorbidities and resistant hypertension does not appear to influence the outcomes of autologous DC administration in this patient population.

## 4. Discussion

This study showed that ADC values did not correlate with the severity of DKD. This result contrasts previous studies showing a significant correlation between mean renal ADC values and various degrees of severity of chronic kidney disease (CKD). Prior studies generally found that ADC values decreased as the severity of CKD increased (Çakmak et al., 2014a [8]). However, no similar pattern was found in this study. This study differentiated the degree of CKD based on grades 1–2 and 3. The lack of correlation between ADC and GFR is similar to the meta-analysis by Liu et al., who explained that although ADC was able to differentiate between CKD grade 1–2 and normal kidney, there was no significant difference in ADC values between CKD grade 1–2 and grade 3. Liu et al. found significant heterogeneity when comparing ADC between grade 3 and grade 1–2 CKD, possibly due to variations in imaging parameters, ROI (Region of Interest) determination methods, and b values used in various studies. In addition, pathological changes in grade 1–2 CKD may still be similar to those in grade 3 CKD, such as fibrosis and decreased perfusion that have not led to significant differences in ADC values. This explains why the correlation between ADC and GFR is inconsistent, especially in the early to intermediate stages of CKD [18].

The results showed that autologous dendritic cell administration did not significantly change ADC, TGF-β, and ICAM-1 parameters. However, more detailed findings showed a dynamic relationship between TGF-β and ICAM-1 before and after autologous dendritic cell administration and a significant correlation between ICAM-1 and ADC in specific subgroups. Before the administration of autologous dendritic cells, it was found that TGF-β and ICAM-1 had a significant positive correlation. This indicates that in patients with DKD who have not received the intervention of autologous dendritic cell administration, TGF-β plays a vital role in changes in ICAM-1 levels. This positive correlation suggests that increased TGF-β levels are likely to be associated with increased ICAM-1 activity, which plays a role in the inflammatory process and fibrosis in the kidney. This was revealed by Chen et al. in that TGF-β is a regulator of the renal fibrosis process in DKD. TGF-β will promote excessive extracellular matrix accumulation through collagen and fibronectin production. Although not directly related to ICAM-1, TGF-β-induced fibrosis alters renal hemodynamics, increasing ICAM-1 expression [19]. However, TGF-β has a paradoxical role in inflammation, with its anti-inflammatory function. This is evidenced when the inhibition of TGF-β will increase inflammation [20]. Increased TGF-β1 plays a role in converting naive T cells into regulatory T cells (Treg), which can suppress excess immune responses and reduce inflammation, thus helping to repair kidney damage in diabetic kidney disease (DKD). In addition, TGF-β1 can reduce leukocyte and macrophage infiltration and suppress the production of pro-inflammatory cytokines such as IL-1β and TNF-α, directly contributing to reducing inflammation and slowing the progression of DKD [21].

In line with these findings, a subgroup analysis based on gender found that the increase in ICAM-1 levels in female subjects was statistically significant. Research by Hwang et al. also explained that women or people with diabetes will have significantly higher ICAM-1 levels than men or people without diabetes [22]. A significant improvement also followed these results in serum creatinine levels. This finding might be caused by estrogen’s protective role. Estrogen protects against hypertension by reducing sympathetic activity, enhancing baroreflex sensitivity, lowering oxidative stress, increasing nitric oxide production, and balancing the renin–angiotensin–aldosterone system. Through estrogen receptors in brain regions like the subfornical organ, paraventricular nucleus, and rostroventrolateral medulla, it modulates neurogenic and inflammatory pathways to lower blood pressure [23].

This finding is interesting because increased ICAM-1, usually associated with increased inflammation, is followed by improved renal function, reflected in decreased serum creatinine. This could reflect the fact that in the female subgroup, the response to autologous dendritic cell administration is different from that of men, where increased ICAM-1 levels may not only reflect increased adverse inflammation but also a more adaptive or regulative immune response to inflammation. This finding is also consistent with the study by Bui et al., who stated that, based on current evidence, ICAM-1 has a role in inflammation subsidence and tissue repair. This is achieved by modulating the efferocytosis function of macrophages for repair. Although there is no evidence of epithelial and endothelial tissue repair in the kidney, the effect of tissue healing in the colon, skin, and cornea hints at the possible influence of ICAM-1 on improving kidney function due to DKD [24]. Endothelial damage is a key component in the pathogenesis of DKD, contributing significantly to its progression. As part of the pathological features, endothelial cell injury occurs alongside glomerular mesangial expansion, basement membrane thickening, podocyte loss, and nodular glomerulosclerosis. This endothelial damage is not merely an incidental finding but plays a central role in the development of the disease [25]. In the early stages, it leads to tubular hypertrophy, which eventually progresses to interstitial fibrosis and tubular atrophy. This progression highlights the critical role of endothelial dysfunction in driving the pathological changes seen in DKD [26].

This study has several limitations that should be considered. The small sample size of 22 patients limits the generalizability of the findings, and the quasi-experimental design without a control group reduces the ability to draw firm conclusions on the treatment’s efficacy. While changes in biomarkers like TGF-β and ICAM-1 were observed, no significant alterations in ADC values or kidney function were found, suggesting that more refined methods or longer follow-up may be necessary to detect meaningful effects. Additionally, the focus on gender differences and comorbidities indicates the need for larger, more diverse studies to validate these findings.

## 5. Conclusions

This study evaluated the impact of autologous dendritic cell administration on markers of kidney function and inflammation in diabetic kidney disease (DKD). The results showed no significant changes in ADC values, TGF-β levels, or overall kidney function across the patient cohort. However, a gender-specific response was observed, with significant changes in ICAM-1 levels in female participants, suggesting a possible adaptive immune response. This finding warrants further investigation into the role of gender in immune modulation and treatment response. While this study identified promising trends, such as potential improvements in renal function in females, the absence of significant overall changes in key biomarkers and kidney function suggests that the further optimization of treatment protocols is necessary.

Looking ahead, future research should aim to address the limitations of this study, including the small sample size and the lack of a control group, by conducting larger, randomized controlled trials. These trials should incorporate diverse patient populations, accounting for variables such as comorbidities, medication use, and baseline immune status, which may influence the effectiveness of dendritic cell therapy. Additionally, exploring the long-term effects of dendritic cell administration and its potential for slowing disease progression in DKD is essential. Further studies should also investigate the mechanisms underlying the observed gender differences and explore potential combinatory therapies that might enhance the therapeutic impact of dendritic cells, ultimately contributing to more personalized and effective treatment strategies for DKD.

## Figures and Tables

**Table 1:** Subjects’ baseline characteristics.

Baseline Characteristics		
**Number of subjects**		22
Gender, n (%)	Men	9 (41)
	Women	13 (59)
Age, mean ± SD		64 ± 7.8
Comorbidities, n (%)	Hypertension	20 (90.9)
	Heart Disease	10 (45.5)
	Stroke	3 (13.6)
	Neuropathy	14 (63.6)
BMI categories, n (%)	Underweight (≤17.5 kg/m^2^)	1 (4.5)
	Normal weight (17.50–22.99 kg/m^2^)	6 (27.3)
	Overweight (23.00–27.99 kg/m^2^)	9 (40.9)
	Obesity (≥28 kg/m^2^)	6 (27.3)
Types of anti-diabetics consumed, n (%)	Sulphonylurea	6 (27.3)
	Biguanide	5 (22.7)
	Dipeptidyl peptidase-4 inhibitors	4 (18.2)
	Sodium-glucose cotransporter-2 inhibitors	8 (36.4)
	Insulin	17 (77.3)
Anti-hypertensive types consumed, n (%)	Angiotensin receptor blocker	15 (68.2)
	Angiotensin-converting enzyme inhibitors	3 (13.6)
	Calcium channel blockers	16 (72.7)
	Beta-blockers	9 (40.9)

**Table 8:** ADC values, serum creatinine levels, ICAM-1, and TGF-β changes in heart disease group and resistant hypertension group.

Variables	Time Point	Group					
		Heart Disease Comorbidity			Resistant Hypertension		
		Absent (*n* = 12)	Present (*n* = 10)		Absent (*n* = 14)	Present (*n* = 8)	
		Mean ± SD		*p*-Value ^1^	Mean ± SD		*p*-Value ^1^
ADC ^a^ (mm^2^/s)	Pre	1.680 (0.229)	1.683 (0.891)	0.895	1.668 (0.381)	1.806 (0.611)	0.539
	Post	1.757 (0.288)	1.1517 (0.308)		1.637 (0.287)	1.664 (0.767)	
	*p*-value ^2^	0.480	0.445		0.510	0.208	
ICAM-1 ^b^ (ng/mL)	Pre	329.97 ± 27.87	312.33 ± 19.44	0.578	338.25 ± 21.68	293.42 ± 27.53	0.300
	Post	334.94 ± 16.97	331.26 ± 16.23		339.89 ± 14.41	321.67 ± 19.96	
	*p*-value ^2^	0.767	0.333		0.893	0.311	
TGF ^b^ (ng/mL)	Pre	44.67 ± 47.14	36.422 ± 28.41	0.465	45.47 ± 39.70	3.29 ± 2.56	0.502
	Post	45.51 ± 40.78	40.54 ± 43.75		46.67 ± 38.98	3.73 ± 3.87	
	*p*-value ^2^	0.796	0.193		0.667	0.282	
Creatinine ^b^ (mg/dL)	Pre	1.27 ± 0.15	1.59 ± 0.17	0.544	1.56 ± 0.15	1.16 ± 0.17	0.468
	Post	1.24 ± 0.17	1.50 ± 0.14		1.52 ± 0.13	1.06 ± 0.19	
	*p*-value ^2^	0.636	0.200		0.595	0.199	

^a^ Result are presented as median (interquartile range), as the data were non-normally distributed; ^b^ the data were normally distributed; ^1^ Independent Samples *t*-test (for normally distributed data) or Mann–Whitney test (for non-normally distributed data) used to assess the difference between groups; ^2^ Paired *t*-test (for normally distributed data) or Wilcoxon (for non-normally distributed data) used to compare between pre- and post-autologous DC administration.

## Data Availability

All data are available upon request.
